# Microglia in Alzheimer's Disease: A Role for Ion Channels

**DOI:** 10.3389/fnins.2018.00676

**Published:** 2018-09-28

**Authors:** Laura Thei, Jennifer Imm, Eleni Kaisis, Mark L. Dallas, Talitha L. Kerrigan

**Affiliations:** ^1^Reading School of Pharmacy, University of Reading, Reading, United Kingdom; ^2^University of Exeter Medical School, University of Exeter, Exeter, United Kingdom

**Keywords:** microglia, Alzheimer's disease, ion channel, stem cells, iPSCs

## Abstract

Alzheimer's disease is the most common form of dementia, it is estimated to affect over 40 million people worldwide. Classically, the disease has been characterized by the neuropathological hallmarks of aggregated extracellular amyloid-β and intracellular paired helical filaments of hyperphosphorylated tau. A wealth of evidence indicates a pivotal role for the innate immune system, such as microglia, and inflammation in the pathology of Alzheimer's disease. The over production and aggregation of Alzheimer's associated proteins results in chronic inflammation and disrupts microglial clearance of these depositions. Despite being non-excitable, microglia express a diverse array of ion channels which shape their physiological functions. In support of this, there is a growing body of evidence pointing to the involvement of microglial ion channels contributing to neurodegenerative diseases such as Alzheimer's disease. In this review, we discuss the evidence for an array of microglia ion channels and their importance in modulating microglial homeostasis and how this process could be disrupted in Alzheimer's disease. One promising avenue for assessing the role that microglia play in the initiation and progression of Alzheimer's disease is through using induced pluripotent stem cell derived microglia. Here, we examine what is already understood in terms of the molecular underpinnings of inflammation in Alzheimer's disease, and the utility that inducible pluripotent stem cell derived microglia may have to advance this knowledge. We outline the variability that occurs between the use of animal and human models with regards to the importance of microglial ion channels in generating a relevant functional model of brain inflammation. Overcoming these hurdles will be pivotal in order to develop new drug targets and progress our understanding of the pathological mechanisms involved in Alzheimer's disease.

## Introduction

Alzheimer's disease (AD) is the most prevalent neurodegenerative disorder and accounts for approximately 60–80% of all dementia cases worldwide (Alzheimer'sstatistics, 2016). Initial studies focussed on trying to identify a genetic basis to the disease (Gatz et al., 2006). Although some AD cases are caused by defined mutations in one of three genes (APP, PSEN1 and PSEN2) these account for fewer than 10% of all cases and occur before 65 years of age. The majority of cases are sporadic, have no defined etiology and occurs at or after a mean age of 65. Our understanding has progressed through evidence obtained from large cohort studies identifying genetic variants which are associated with and potentially result in the late onset form of AD (LOAD). These genome wide association studies (GWAS) have demonstrated that LOAD is a multifactorial disease with many different genes and single nucleotide polymorphisms contributing to disease onset (Gatz et al., 2006). The most strongly associated gene with LOAD is Apolipoprotein E (APOE), which encodes a polymorphic glycoprotein that is involved in cholesterol and other lipid transport (Poirier, 2005) alongside tissue repair (Huang, 2010) and neuronal growth (Nathan et al., 1994). There are three isoforms of APOE, ε2, ε3, and ε4 that all correspond to cysteine to arginine substitutions at the amino acid positions 112 and 158 (Zlokovic, 2013). The ε4 variant confers increased risk of developing LOAD, and each additional copy of the ε4 allele lowers the mean age of onset (Corder et al., 1993). Neurodegenerative diseases such as AD were traditionally considered to be ‘'neurocentric,” however recent findings are challenging this view, implicating glia as primary targets. GWAS studies reveal there have been a number of single nucleotide polymorphisms that are associated with AD which reside in genes involved in microglial biology. These include common variants such as CR1 (complement receptor 1), CD33 (sialic acid binding Ig-like lectin 3), CLU (clusterin), ABCA7 (ATP-binding cassette, sub family A, member 7), MS4A (membrane-spanning 4-domain family, subfamily A) and EPHA1 (ephrin type-A receptor 1) (Bertram et al., 2008; Harold et al., 2009; Hollingworth et al., 2011; Naj et al., 2011; Lambert et al., 2013; Zhang et al., 2013), and also more rare coding variants in genes such as TREM2 (triggering receptor expressed on myeloid cells 2) (Guerreiro et al., 2013; Jonsson et al., 2013). TREM2 is a cell surface receptor of the immunoglobulin superfamily that is expressed on microglia (reviewed by Colonna and Wang, 2016). Several variants within TREM2 appear to significantly increase the risk of developing AD (Jin et al., 2014; Song et al., 2017), in particular rs75932628, an SNP that confers an arginine to histidine change at amino acid 47 (R47H) (Guerreiro et al., 2013; Jonsson et al., 2013). Although TREM2 polymorphisms are associated with a risk of late-onset AD (Guerreiro et al., 2013), their role in neurodegenerative diseases is controversial. Indeed, recent evidence proposes that the TREM2-APOE pathway induces a microglia phenotypic switch from a homeostatic to neurodegenerative phenotype (Krasemann et al., 2017). One of the main functions of TREM2 is regulating microglial phagocytosis (Hsieh et al., 2009), and as a ligand for TREM2 in microglia, APOE binds to dead neurons and increases Trem2-mediated phagocytosis (Atagi et al., 2015). Interestingly, Kleinberger et al. (2014) showed that missense mutations in TREM2 resulted in impaired phagocytic activity with a reduced level of soluble TREM2 in cerebrospinal fluid (CSF) of AD patients. Indeed TREM2 deficiency has been shown to alter microglial function in both primary microglial cultures and in mouse models of AD where a decrease in plaque-associated microglia are observed alongside an increase in apoptosis of both resting and activated microglia and reduced phagocytosis (Ulrich et al., 2014; Jay et al., 2015, 2017). These findings suggest that the role of TREM2 in modulating inflammation may be more complex than previously appreciated and may be dependent on the cell type in which it is expressed and the inflammatory context in which it is studied. For a more in depth discussion we refer the reader to the following very comprehensive review articles (Colonna and Wang, 2016; Ulrich et al., 2017; Li and Zhang, 2018).

Microglia are thought to regulate the degree of Aβ deposition by phagocytosis with potentially protective impact on AD progression (Lee and Landreth, 2010). One striking feature of the behavior of microglia in the AD brain is their marked clustering around fibrillar Aβ deposits and they adopt a polarized morphology with hypertrophic processes extending toward plaques (Condello et al., 2015). This aids as a protective physical barrier mechanism through which the Aβ fibrils cannot extend, promoting the formation of highly compact plaque micro regions that have minimal affinity for soluble Aβ_1−42_ (Condello et al., 2015; Yuan et al., 2016). Conversely, areas not covered by microglia processes display “hotspots” with very high soluble Aβ_1−42_ affinity, leading to markedly concentrated protofibrillar Aβ42 plaque regions (Condello et al., 2015). These “hotspots” are neurotoxic given that adjacent axons develop a greater extent of dystrophy compared to those covered by microglia (Yuan et al., 2016).

On the other hand, most studies in TREM2- deficient AD-like mice have shown reduced number of microglia around Aβ plaques (Jay et al., 2015; Wang et al., 2015). Similar reports suggest that in R47H human mutants, microglial processes were also unable to form a robust barrier, resulting in a decreased Aβ fibril compaction (Yuan et al., 2016). With the decrease in microglial number, there are less compact Aβ fibrils and a higher ratio of Aβ_1−42_ plaques (Yuan et al., 2016; Ulland et al., 2017), therefore a deficient rather than an exacerbated microglial response could give rise to the development of sporadic AD. Once activated by pathological triggers, like neuronal death or protein aggregates, microglia extend their processes to the site of injury, migrate to the lesion and initiate an innate immune response (Heneka et al., 2015). Mounting evidence from polymorphisms linking microglial dysfunction to AD could have a causal role in disease onset and progression and are not just a consequence of neuropathological hallmarks that are characteristic of AD.

## The innate immune system in AD

Of increasing interest is the involvement of the innate immune system in AD, particularly the role of microglia. Microglia are the resident immune cells in the brain and spinal cord, and play important roles in neurodevelopment, immune surveillance, disease and homeostasis (Nayak et al., 2014). Unlike neurons and other glial cell types, microglia are of haematopoietic lineage, arise early during development (Hutchins et al., 1990), and are derived from erythromyeloid progenitors (EMPs) in the yolk sac (Ginhoux et al., 2010).

Microglia can exist in several morphological/phenotypic states depending on the environment they are in or the factors they are stimulated by. From a highly processed state, the microglia become more amoeboid with increased numbers of intracellular vesicles in preparation for engulfment of foreign particles. These differential states have been termed accordingly as “classical activation,” “alternative activation,” and “acquired deactivation” (Colton, 2009; Colton and Wilcock, 2010). Previous studies defined these states as separate from one another, a profiling index of M1 or M2 phenotyping suggesting a pro- or anti-inflammatory state respectively. More recently it has become more apparent that this is derived from the idea that microglia are central macrophages and so must follow by the same “kill or cure” switch seen in these cell types. However, microglia can exist in multiple phases with the same cell producing markers of both pro- and anti- inflammatory components depending on stimulus. Usage of M1/M2 profile terminology fails to capture the heterogeneity of microglia which is a vital to their local and global physiological responses (Mosser et al., 2017).

**Figure 1:**
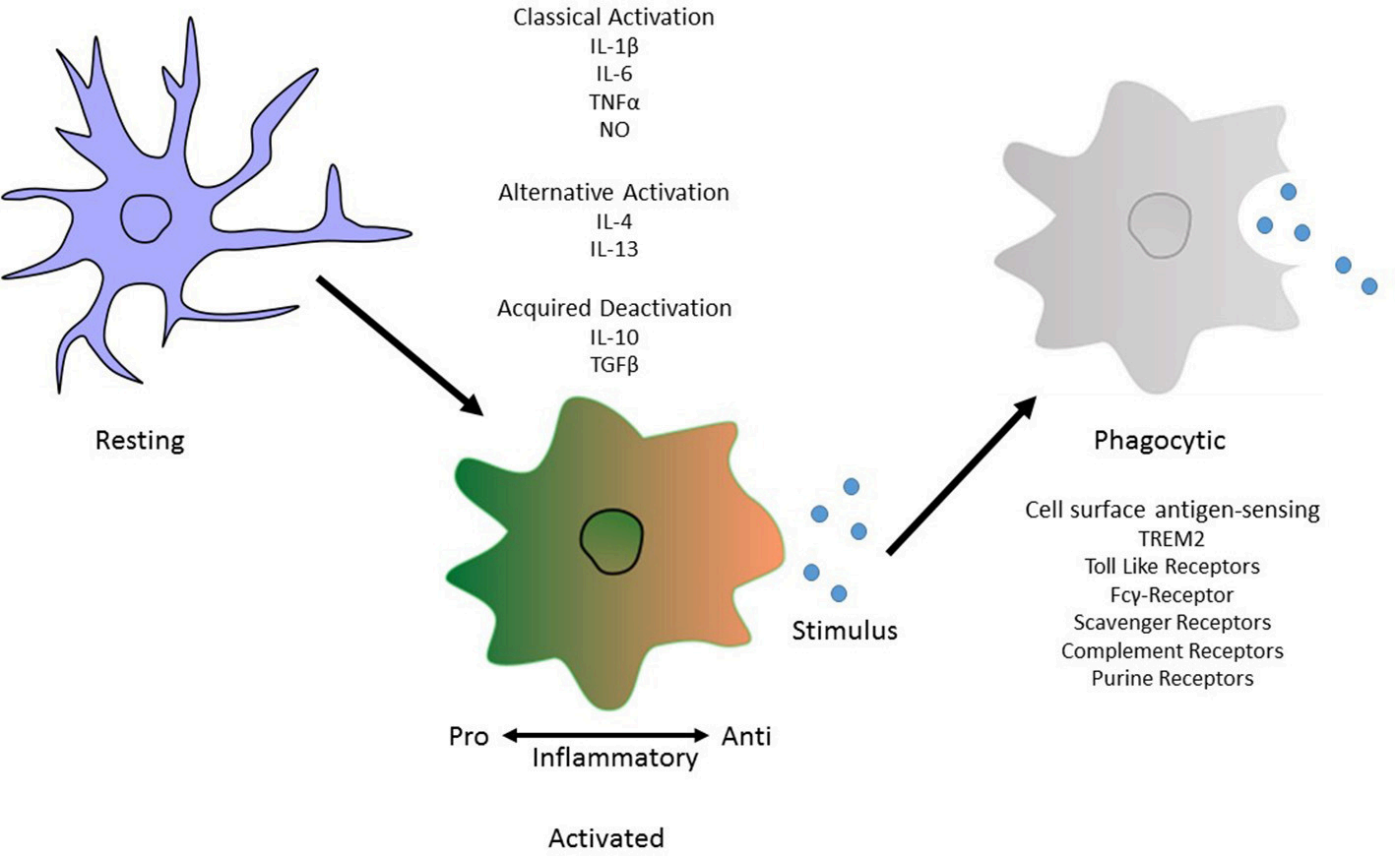
Morphological phenotype of Microglia. An illustration depicting the different phenotypic states. These are “classical activation,” “alternative activation,” and “acquired deactivation.” Classical activation, otherwise considered to be the M1 phenotype and so is pro-inflammatory, is stimulated by IFN-γ and is associated with the production of cytokines such as TNF-α and IL-1β. Subsequently the alternative activation, or M2 phenotype, defined by the release of anti-inflammatory cytokines IL-4 and IL-13. The third activation phenotype, acquired deactivation, is thought to be a subtype of the M2 phenotype, releases IL-10 and TGF-β. Activation does not inclusively mean a phagocytic phenotype in microglia. For this to occur, antigen sensing receptors are made available on the cell surface to allow pathogen recognition. In Alzheimer's disease, the best known of these is TREM2 but others include Toll-like receptors and members of the complement system.

Classical activation, considered to be pro-inflammatory, is stimulated by IFN-γ and is associated with the production of cytokines such as TNF-α and IL-1β and nitric oxide production (Li et al., 2004; Block et al., 2007). On the other hand, alternative activation, is defined by the release of anti-inflammatory cytokines IL-4 and IL-13 and arginase 2. This results in gene expression to promote tissue repair and extracellular matrix reconstruction (Ponomarev et al., 2007; Colton, 2009). Acquired deactivation, is mainly seen in the presence of apoptotic cells and is characterized by the release of IL-10, TGF-β, IL-6, and CSF1 and the production of scavenger receptors (Sawada et al., 1999; Colton, 2009; Colton and Wilcock, 2010; Saijo and Glass, 2011). Microglial phagocytosis relies on specific receptors expressed on the cell surface and their downstream signaling pathways to instigate engulfment of harmful particulates (Figure 1).

Microglia mediate the innate immune response of the brain and are involved in the phagocytosis and clearance of debris, pathogens, and toxins. Their dysfunction and increased Aβ accumulation is universal to AD patients and not just those with familial APP mutations. This suggests that Aβ build-up is due to poor clearance and not APP proteolysis. Microglia will secrete both pro- and anti-inflammatory factors, which can either be beneficial or detrimental in neurodegenerative diseases. Here exists extensive literature showing that inflammation is integral to AD progression, facilitating Aβ deposition, neuronal loss and cognitive deficits. Brains from AD patients and those from murine models of Aβ pathology uniformly display high expression of pro-inflammatory cyto- and chemo- kines including TNFα, IFNγ, IL-1β, and IL-6 (Zheng et al., 2016). IL-1β and TNFα can impair neuronal function by suppression of long-term potentiation of synaptic transmission (LTP) (Rowan et al., 2007). Multiple interactions as well as elevated expression of additional cytokines/chemokines and innate immune receptors favor a pro-inflammatory activation state in AD.

Accumulating evidence demonstrates that inflammasomes, which cleave precursors of interleukin-1β (IL-1β) and IL-18 to generate their active forms, play an important role in the inflammatory response in the CNS and in AD pathogenesis. The inflammasome is an inducible, high molecular weight, protein complex consisting of the antigen sensor protein NLRP3, adaptor protein ASC, and pro-caspase 1 (Heneka et al., 2015). The complexing of these three components results in cleavage of caspase 1 and instigates a cascade of pro-inflammatory cytokine activation of the IL-1b family. In murine mutants where APP/PS1 was crossed with NLRP3-/- mice, a decrease in cC1 and IL-1β is observed (Heneka et al., 2013).

Conversely an anti-inflammatory profile of microglia also contributes to Aβ pathology. In murine models where IL-10 was either knocked down or knocked out in the APP/PS1 model, a decrease in Aβ load, increases phagocytosis and reduces microglial APOE expression was observed (Chakrabarty et al., 2015). Further studies showed that this was due to preventing downstream pathways involving Jak1/Stat3 and consequential transcription factor activity (Guillot-Sestier et al., 2015). Additionally, primary microglia treated with fibrillar Aβ_1−42_ and recombinant IL-10 showed that fibrillar Aβ_1−42_ is prevented from inducing a pro-inflammatory response of cytokine release including CCL5, CXC10, and TNFα, suggesting a push to an anti-inflammatory profile (Chakrabarty et al., 2015).

Therefore, it is pertinent to think that the Aβ activates microglia and results in an innate immune response. Indeed, it has been shown that exposure of microglia to fibrillar Aβ by CD36, a class B scavenger receptor (Coraci et al., 2002), causes the formation of a heterodimer of the TLR4 and TLR6 through NF-κB signaling (Stewart et al., 2010). However, on deletion of MyD88, an adaptor protein essential for downstream TLR signaling, there was a significant decrease in both Aβ load and microglial activation in APP/PS1 mice (Lim et al., 2011). Despite this the MyD88 deletion only resulted in minor improvements in cognitive functions (Lim et al., 2012).

Microglial activation by Aβ does not necessarily only occur after Aβ deposition but can also occur before plaques are even formed. Maezawa and colleagues have shown that nanomolar concentrations of Aβ oligomers activated microglia and that they required another scavenger receptor, SR-A, and the Ca^2+^-activated potassium channel KCa3.1 (Maezawa et al., 2011). Another group has also shown microglial activation precedes Aβ aggregation in APP[V717I] transgenic mice and that this coincides with increased BACE1 activation (Heneka et al., 2005).

Intracellular neurofibrillary tangles of hyperphosphorylated tau are another pathological hallmark of AD. However, the exact mechanisms which lead to the hyperphosphorylation of tau are still unclear. Previously, it has been demonstrated that neuro-inflammation positively correlates with tau aggregation, hyperphosphorylation and neurodegeneration in several models (Sheng et al., 1997; Sheffield et al., 2000; Bellucci et al., 2004, 2011; Ikeda et al., 2005; Yoshiyama et al., 2007).

Microglial activation also precedes tau pathology in the P301S tauopathy model (Yoshiyama et al., 2007). In the triple transgenic model of AD, lipopolysaccharide administration significantly increased tau phosphorylation through toll like receptor 4 signaling (Kitazawa et al., 2005). Interestingly, one paper has demonstrated that microglia may be involved in the propagation of tau pathology through non-synaptic transmission in mammals (Asai et al., 2015). Asai et al. (2015) used two different tau mouse models to show that tau propagation is mediated through microglia which phagocytose tau-positive neurons or synapses and secrete tau protein in exosomes, efficiently transmitting tau to neurons. They also demonstrated that this propagation is sensitive to microglial depletion and inhibition of nSMase2 activity. On the other hand, significant ablation of microglia in a mouse model of amyloidopathy indicated that Aβ formation, maintenance and associated neuritic dystrophy was not depended on microglia (Grathwohl et al., 2009). Interestingly, (Krabbe et al., 2013) reported that Aβ may directly affect microglial function. This *in vivo* study detected a significant inverse correlation between Aβ plaque burden and microglial phagocytic activity (Krabbe et al., 2013). They found that microglial dysfunction develops early during AD in an Aβ-dependent fashion and can be restored by interventional anti-Aβ approaches, such as Aβ vaccination (Krabbe et al., 2013).

## Microglia physiology and ion channels

**Figure 2:**
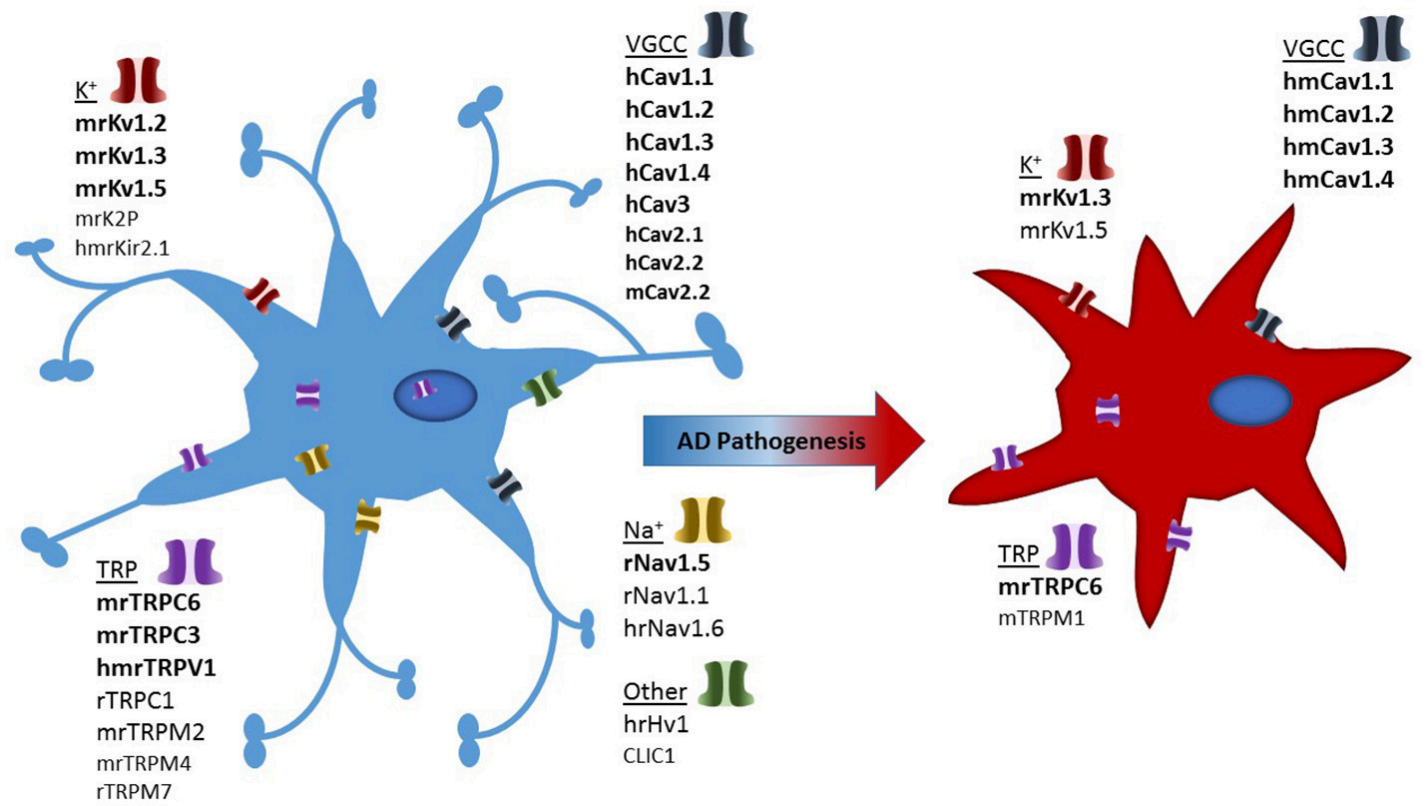
Illustration depicting presence of ion channels observed in microglial models and which of these have confirmed activity in Alzheimer's disease associated microglia. Strength of evidence is depicted in bold to un-bold text. In addition each channel is pre-fixed with the species in which they have been investigated: h, human; m, mouse; r, rat.

Studies have highlighted the importance of microglia in brain ionic homeostasis (Annunziato et al., 2013; Szalay et al., 2016; Shibata and Suzuki, 2017). For example, depletion of microglia results in the loss of potassium chloride induced neuronal depolarisation (Szalay et al., 2016) and the microglia KCa3.1 channel has been proposed as a valid therapeutic target for modulating cortical spreading depression (Shibata and Suzuki, 2017). Therefore ion channels and transporters, regulating ionic flux, are essential regulators of a variety of microglial functions, including proliferation, morphological changes, migration, cytokine release and reactive oxygen species production (Schilling and Eder, 2015). Ion channel expression in microglial cells is tightly regulated, with the expression of most ion channel types noticeably depending on the cells' functional state (Eder, 1998, 2005, 2010; Kettenmann et al., 2011). Despite being non-excitable cells, the plethora of voltage-gated ion channels present in microglia suggests they play a prominent role in both physiological as well as pathological states. Brain inflammation is a characteristic of AD and numerous studies have demonstrated that microglia can directly interact with neurons to induce inflammation (Hashioka et al., 2012). Due to this interaction, the study of microglial ion channels may shed light on brain inflammation seen in neurodegenerative diseases such as AD (Silei et al., 1999). In this review, we have summarized the most prominent ion channels involved in microglial cells which may contribute to AD pathology, as demonstrated in Figure 2.

## Potassium channels

Potassium channels are present in all cells within the body and have many diverse functions. In particular, they are capable of regulating cell excitability and influence action potential waveform. To identify therapeutic targets to modulate microglial activation, numerous studies are addressing the contributions of several K^+^ channels. Based on both their structural and functional properties, K^+^ channels have been subdivided into specific families. They have transmembrane helices (TMs) spanning the lipid bilayer (Kuang et al., 2015). The largest of these consist of K^+^ channels that are activated by membrane depolarisation, with subsequent families consisting of channels that are activated by altered intracellular Ca^2+^ ions and others that are constitutively active. Based on the structure and function, the channels are categorized into three major classes: the voltage-gated (Kv) (six TMs), inwardly rectifying (Kir) (two TMs), and tandem pore domain (K2P) (four TMs) channels (Kuang et al., 2015). K^+^ channels are particularly important in microglia since their activation can induce membrane hyperpolarisations, which are essential for driving Ca^2+^ influx through inward rectifying Ca^2+^-Release-Activated-Ca^2+^ channels (CRAC) (Kraft, 2015; Nguyen et al., 2017a) ATP-activated P2X receptors (Burnstock, 2015) and other Ca^2+^-permeable cation channels (Kettenmann et al., 2011).

### Voltage-gated potassium channels

Kv channels form an exceedingly diverse group, their structure consists of six TMs, of which the first four helices (S1–S4) form the voltage sensor domain (VSD) (Jiang Y. et al., 2003; Long et al., 2007). The last two helices (S5–S6, corresponding to the outer and inner helices in KcsA, respectively) form the pore-forming domain. The VSD senses the membrane potential alteration, and is followed by a conformational change that is coupled to gate the pore-forming domain (Long et al., 2005). In more general terms Kv currents can be classified into showing A-type (inactivating) or delayed rectifier behavior (non-inactivating). The Kv channels present in microglia to date have been summarized in Table 1 and mainly comprises of delayed rectifier Kv channels.

**Table 1:** Microglia ion channels and their functionality after cell activation.

**Channel type**	**Subunit**	**Expression and modulation**	**Proposed functions**	**Models**	**References**
Voltage-gated Calcium Channels (VGCC)	N-type Ca_V_2.2 Possible L-type (Cav1.X)	Very low expression levels at both protein and mRNA level	Cav2.2 control chemokine release from microglia Cav1.2 and 1.3 activity results in proliferation, increased intracellular Ca^2+^ with Aβ, microglial survival and pro-inflammatory action	Rat, mouse and Human primary cultures BV2 cell lines	Silei et al., 1999 Hashioka et al., 2012 Nicoletti et al., 2017 Espinosa-Parrilla et al., 2015
Inwardly rectifying potassium current (K_ir_)	K_ir_2.1	Generally expressed in activated microglia; very low densities in resting microglia Modulated by intracellular factors, such as, G-proteins, intracellular calcium, pH and protein kinase C (PKC) LPS reduces or enhances current density in cultured murine microglia.	Sets a negative resting membrane potential (RMP)	Rat, mouse and primary and secondary cultures BV2 cell line Adult mouse brain slices	Schilling et al., 2000 Eder et al., 1999 Draheim et al., 1999 Boucsein et al., 2003 Kettenmann et al., 1993, 2011; Nörenberg et al., 1994a; Fischer et al., 1995; Chung et al., 1999; Franchini et al., 2004
Delayed outwardly rectifying potassium current (K_dr_)	K_v_1.3 K_v_1.5 To a lesser extent K_v_1.1 K_v_1.2	Transiently up-regulated upon stimulation with LPS interferon-β, TNF-α, TGF-β, GM-CSF, pH, Aβ, prostaglandin E2 receptor activation. Hypoxic insults increase Kv1.2 expression in adult microglial cells	Re-establishing a negative RMP during membrane oscillations Both K_v_1.3 and K_v_1.5 play a role in proliferation and ROS production K_v_1.3 is involved the respiratory burst K_v_1.5 is involved in NO release K_v_1.1 and K_v_1.2 involved LPS-induced release of TNF-α, interleukin-1β, endothelins and NO	Rat and mouse primary cultures Human brain slices and primary cultures	Nörenberg et al., 1994a; Schilling et al., 2000; Fordyce et al., 2005 Jou et al., 1998; Kotecha and Schlichter, 1999; Khanna et al., 2001 Pannasch et al., 2006; Eder, 1998 Chung et al., 1999 Eder and Heinemann, 1996
Ca^2+^-dependent potassium channels (KCa)	Large conductance: KCa1.1 Small conductance: KCa2.1 KCa2.2 KCa2.3 KCa3.1	KCa2.3 channels are predominantly expressed in both cultures and healthy striatum tissue; LPS treatment or ischemic insult increased the level of expression.	Blocking KCa2.2/KCa2.3 with CyPPA reduces cytokine release Migration was inhibited when KCa1.1 was blocked with charybdotoxin and clotrimazole Small conductance channels are linked to NO release, MAPK signaling and respiratory burst Inhibition of KCa2.3 affected microglial activation and reduced microglial neurotoxicity.	Calf, rat, mouse, human, adult, and new born rat primary cultures and brain slices	Walz et al., 1993 McLarnon et al., 1997; McLarnon et al., 2001; Bordey and Spencer, 2003 Khanna et al., 2001 Spranger et al., 1998 Papavlassopoulos et al., 2006; Stock et al., 2006; Kaushal et al., 2007
Two-Pore Domain Potassium Channel (K2P)	K2P13.1 (THIK-1)	High RNA expression in resting microglia Age-dependent decrease of mRNA	Maintaining RMP. Mediates release of the pro-inflammatory cytokine interleukin-1β from activated microglia Regulates microglial ramification	Rat hippocampal brain slices and primary cultures	Madry et al., 2018
Voltage-gated Na^+^ channels	Na_v_1.1, Na_v_1.5 Na_v_1.6	Na_v_1.6 expression correlates with the transition between resting and activated microglia in EAE Protein and mRNA for Na_v_1.6 were detected in EAE mice spinal cord and optic nerve, as well as in MS-affected human spinal cord.	Phagocytosis and cell volume regulation Treatment of LPS-activated microglia with TTX or phenytoin reduced IL-α1, IL-1β, and TNF-α secretion Decreased motility responses to ATP Phenytoin reduced microglial activation in EAE model	Rat primary cultures	Craner et al., 2005 Korotzer and Cotman, 1992 Nörenberg et al., 1994b Schmidtmayer et al., 1994 Eder, 2005 Black et al., 2009
Transient Receptor Potential channels (TRP)	TRPC1, 3, 6 TRPM2, 4 TRPV1, 2 and to a lesser extent other TRPs	Low expression of RNA in resting microglia. Transiently up-regulated upon stimulation with LPS, ATP, or BDNF	Activation of TRPs contributes to microglial Ca^2+^ signaling and are linked to IL-6 release, NO, TNFα associated initiation of microglial cells death Contribute to the regulation of transcription factors' function, including NFkB, NRF2, and AP-1, cytokine production, cell proliferation, activation, apoptosis and oxidative stress	Rat and mouse primary cultures BV-2 cell lines Human primary cultures	Sun et al., 2014 Mizoguchi et al., 2014 Liu et al., 2017 Huang et al., 2017 Sappington and Calkins, 2008 Schilling and Eder, 2011
Proton channels	H_v_1 voltage-activated H^+^ channels	Unknown	Single-channel conductance fS range High sensitivity to extracellular pH; associated with generation of respiratory burst Disruption of cytoskeleton leads to a 50% reduction in H^+^ current amplitude Cell swelling potentiates H^+^ currents.	Mouse, rat and human primary cultures	Eder et al., 1995; Klee et al., 1998, 1999 Tian et al., 2016 Wu et al., 2012
Chloride channels	CLIC-1	mRNA highly expressed in mammalian microglia channel protein relocation from cytosol to membrane after Aβ	Morphological changes based on Cl^−^ responses to stretch	Rat and bovine primary cultures	Visentin et al., 1995; Schlichter et al., 1996 McLarnon et al., 1995 Skaper et al., 2013 Milton et al., 2008 Paradisi et al., 2008

Kv1.2, Kv1.3, and Kv1.5 transcripts and protein have been detected in both primary rat and mouse microglia (Kotecha and Schlichter, 1999; Khanna et al., 2001; Fordyce et al., 2005; Pannasch et al., 2006; Li et al., 2011). Microglia are widely distributed throughout the brain; however some regions express much higher levels than others (Lawson et al., 1990). The hippocampus, an area particularly affected by AD, is rich with microglia and is especially sensitive to cerebrovascular insults which have been shown to rapidly activate microglia (Wu and Ling, 1998). The reasons for the highly variable expression of Kv channels and the role this plays in non-excitable cells such as microglia are not well understood. It is now known that microglia in culture can express different proteins when compared to microglia *in situ* in brain slices or *in vivo* (Boucsein et al., 2003; Butovsky et al., 2014; Yamasaki et al., 2014; Gosselin et al., 2017). Earlier studies mostly used cultured microglia from enzymatically dissociated tissue, thus removing cell– cell contacts and key secretory products such as growth factors affecting Kv channel expression itself (Kettenmann et al., 1990; Ganter et al., 1992; Draheim et al., 1999). *In vitro* studies are currently the only way to stimulate microglia in isolation in order to elucidate similarities and differences in how different species respond (Lam et al., 2017).

It is becoming more apparent that altered expression of Kv channels could trigger the mechanisms underlying microglial polarity and could characterize these microglial states (Saijo and Glass, 2011; Maezawa et al., 2012). In a study on freshly isolated microglial cells, Kotecha and Schlichter (1999) found both Kv1.3 and Kv1.5, the former being associated with proliferating cells and the latter with non-proliferating cells. This shift in microglial activation also results in changes in the physiological properties of the cells (Kotecha and Schlichter, 1999). Resting microglia express Kv1.5 channels and upon activation and proliferation they upregulate Kv1.3 and down-regulate Kv1.5 channels (Pannasch et al., 2006). Kv1.3 channels migrate to the cell surface while Kv1.5 channels are internalized, making Kv1.3 channels not only functionally relevant but highly susceptible to pharmacological manipulation through selective channel blockers. As we have highlighted, majority of microglial studies use animal models, in particular rodents. Lam et al. (2017) found distinct variability between the different rodent models in expressing different Kv channels. It is also apparent that Kv channel expression of microglial cells in brain slices from juvenile mice (P5-P9) differs to some extent from that of cells in adult mice (Boucsein et al., 2003; Schilling and Eder, 2007; Menteyne et al., 2009; Arnoux et al., 2013, 2014). The passive membrane properties and Kv channel expression of microglial cells undergo substantial changes upon aging (Schilling and Eder, 2015). In comparison with microglia of young adult mice, microglial cells of aged mice are characterized by more negative resting membrane potentials, decreased input resistances and upregulated expression of inward rectifier and outward rectifier Kv channels. Interestingly, the outward rectifier Kv channel current is strongly age-dependent both *in vitro* and *in vivo* (Schilling and Eder, 2015). It is clear from the literature that the way in which we study Kv channel physiology in microglia varies dramatically and depends on the methodology used (Lam et al., 2017).

Further complications include the potential for strain differences in rodents (Becker, 2016), and genetic polymorphisms and epigenetic changes in humans (Boche and Nicoll, 2010). There is considerable debate as to how closely mouse models resemble human responses in inflammatory diseases (Seok et al., 2013; Takao and Miyakawa, 2015). A better understanding of microglial K^+^ channel regulation and expression patterns in neurodegenerative states could also yield targets for drug development using K^+^ channel blockers.

### Voltage-gated potassium channels and AD

Microglia are the key inflammatory cells in AD that mediate neuro-inflammation, and Kv channels are key regulators of microglial function, in particular Kv1.3 (Rangaraju et al., 2015). In animal models of AD, Aβ-induced priming of microglial NADPH oxidative activity depends on Kv1.3 channels, however the exact mechanisms that contribute to this priming is still poorly explored (Kotecha and Schlichter, 1999; Schilling and Eder, 2011). It is thought that the activity of Kv channels lead to membrane hyperpolarization, this Kv1.3 channel-induced membrane hyperpolarisation could enhance Ca^2+^ influx through Transient receptor potential (TRP) channels (see section Calcium Channels; Schilling and Eder, 2011) aiding in the translocation of PKC and therefore leading to NADPH oxidative priming. Franciosi et al. (2006) demonstrated that the broad spectrum Kv channel inhibitor 4-aminopyridine (4-AP) suppressed microglial activation *in vivo* and reduced microglia-induced neuronal death (Franciosi et al., 2006). This inhibition using 4-AP, which also blocks Kv1.3 channels, could attribute to inhibition of microglial priming and subsequent reduction of microglial ROS production, supporting a role for Kv1.3 channels as a therapeutic target in AD (Schilling and Eder, 2011). More recently immunohistochemistry experiments on human brain cortices revealed the presence of Kv1.3 channels in cortical microglia at levels higher than non-AD controls (Rangaraju et al., 2015). This particular study also revealed a “plaque-like” pattern of Kv1.3, suggesting that it may be possible for Aβ to interact with Kv1.3. Interestingly, Aβ_1−42_ oligomers, but not soluble Aβ, accelerate the activation and inactivation kinetics of Kv1.3 channels in lipid bilayers without altering channel conductance (Lioudyno et al., 2012). It is possible that altered channel conductance of Kv1.3 channels could affect calcium fluxes in neurons and microglia, however the relevance of this potential Aβ-Kv1.3-interaction remains to be clarified. Another study by Chung et al. (2001) also confirmed that Aβ was capable of upregulating Kv1.3 as well as the Kv1.5 channel current density. More recently, low levels of soluble oligomeric Aβ have been reported to upregulate primary cultured microglial activity as well as Kv1.3 at transcript and as protein levels (Maezawa et al., 2017). Electrophysiological studies using whole-cell patch clamp also revealed enhanced outward rectifier current, characteristic of homotetrameric Kv1.3 channels. Pharmacological characterization revealed that the currents were sensitive to the Kv1.3 specific blockers ShK-186 (Tarcha et al., 2012), margatoxin (Garcia-Calvo et al., 1993) and the selective Kv1.3 blocker PAP-1 [5-(4-phenoxybutoxy) psoralen (Schmitz et al., 2005). Oligomeric Aβ further induced a significant increase in Kv1.3 current density compared to unstimulated microglia (Maezawa et al., 2017). Following long-term treatment of an APP/PS1 mouse model, the selective Kv1.3 blocker PAP-1 mitigated some key AD-like phenotypes such as reducing Aβ deposition as well as restoring hippocampal synaptic plasticity. The observation that pharmacological targeting of Kv1.3 channels in microglia with the selective inhibitor PAP-1 supports PAP-1 as a promising potential for neuro-immunomodulation therapy and the treatment of neurodegenerative diseases such as AD.

The age-dependent changes in microglial Kv1.3 noted in 5xFAD mice followed a similar trend—initially an age-dependent increase, then a substantial decrease between 10 and 15 months of age. We suspect that these changes in K^+^ channel expression form part of the age-related changes in microglial function, documented by several lines of investigation, such as altered responses to Aβ aggregates or downregulation of “sensome” genes (Hickman et al., 2008; Cameron et al., 2012; Heneka et al., 2013; Hickman and El Khoury, 2013; Johansson et al., 2015) ever, this downregulation is not reflected in a human study in which Kv1.3 expression remains robust in microglia, particularly in the later stages of AD (Rangaraju et al., 2015). More recently transcriptomic data from Rangaraju et al. (2018), revealed that Kv1.3 plays a distinct role in disease-associated-microglia in the 5XFAD mouse model (Rangaraju et al., 2018). It is pertinent to say that the evidence presented here from the existing human and rodent studies, show Kv1.3 could be a therapeutic target even at the late stage of the disease. Similar to what we have previously discussed, it appears that the current transgenic models of AD do not replicate the patterns of microglia activation in human AD. Many potential treatments identified in rodents have failed in human clinical trials. To narrow this translational gap, it is essential to investigate and acknowledge species similarities and differences. With the promises of stem cell therapy and use of iPSCs to model diseases in a dish, pharmacological manipulation on a more directly available human source may reveal further species differences.

### Other potassium channels

Recent evidence has suggested that two-pore domain K^+^ (K2P) channels may play a role in microglia physiology (Madry et al., 2018). Functional investigations provide data to support the involvement of THIK-1 in the cytokine release of microglia *in situ*. This study revealed two functionally and mechanistically distinct modes of microglial motility. THIK-1 regulates microglial ramification, surveillance and interleukin-1β release (Madry et al., 2018). This is the first study of its kind to implicate K2P channels in microglia physiology. Future work will provide a better understanding of its role *in vivo* as well as neuro-inflammatory responses. The impairment of motility of microglial processes that occurs in some pathological conditions, e.g., in models of Alzheimer's disease with Aβ plaque deposition (Koenigsknecht-Talboo et al., 2008; Krabbe et al., 2013; Condello et al., 2015) raises the question of whether the dependence of surveillance on THIK-1 activity can be employed therapeutically for the treatment of AD (Madry et al., 2018). Currently there has been no direct experimental evidence linking THIK-1 to AD.

Another important K^+^ channel that has been shown to play a key role in microglia activation by modulating Ca^2+^ signaling and membrane potential is calcium-activated KCa3.1 (also known as IK1, SK4 or KCNN4) channels (Maezawa et al., 2012). This channel is predominantly expressed in microglia and has been a potential target for both industry and academia as a potential drug target for AD (as reviewed by Maezawa et al., 2012).

The strong inwardly rectifying K^+^ (Kir) channel belong to a family of K^+^ channels that have only two membrane-spanning domains and are responsible for stabilization of the resting membrane potential (V_rest_) near to the K^+^ equilibrium potential (E_K_) (Kettenmann et al., 1990; Tsai et al., 2013). Blocking Kir channels depolarizes the cell and decreases the driving force for inwardly transported Ca^2+^ in microglia. In a study by (Tsai et al., 2013), addition of the AD drug memantine suppressed Kir as well as depolarized the membrane potential of BV-2 cells. This block of Kir2.1 channels could represent one of the important mechanisms underlying its actions on the functional activities of microglial cells. It remains unclear what the *in vivo* function of Kir are, an area showing significant promise for AD.

Interestingly, in the transgenic mouse model of AD (5xFAD) (Wendt et al., 2017) reported that the impairment in phagocytic function of microglia was due to altered purinergic signaling. They found evidence of altered physiological phenotype only of microglia in 5xFAD mice that were located close to Aβ plaques (Wendt et al., 2017). Supporting the idea that functional and pathological alterations of microglia in AD may be a consequence of their association with Aβ plaques. Their detailed study on the 5xFAD model revealed an initial induction of Kir current, followed by subsequent activation of outwardly rectifying currents at a later age. Therefore the induction of Kir current could be considered a first response followed up with outward K^+^ current developing at a later stage of microglial activation, similar to their previous studies (Boucsein et al., 2000; Kettenmann et al., 2011). This data supports the fact that microglia can undergo chronic changes in physiological properties in a disease model over a prolonged period. It appears from the literature that Kv1.3, KCa3.1, and Kir 2.1 inhibitors seem to constitute relatively general anti-inflammatory effects and it could therefore be useful to preferentially target detrimental pro-inflammatory microglia functions associated with neuro-inflammation, such as AD (Nguyen et al., 2017b). A more recent study investigated the effects of Aβ plaque-dependent morphological and electrophysiological heterogeneity of microglia in the AD mouse model, TgCRND8. Plescher et al. (2018) revealed increased K^+^ currents in plaque-associated but not plaque distant microglia. They believe that this electrophysiological heterogeneity is likely to reflect the different functional states of the microglia in TgCRND8 (Plescher et al., 2018). Their finding that outwardly rectifying currents (Kv 1.3) were confined to a subset of plaque associated microglial cells emphasizes the potential of specific ion channel inhibitors to target only specific (i.e., detrimental) subtypes of microglia in AD (Plescher et al., 2018).

## Voltage-gated sodium channels

Sodium voltage channels (NaV) are formed of one pore-α-subunit associated with one/more β-subunits. The α-subunit acts as the “voltage sensor” being activated by changes in membrane potential (Payandeh et al., 2011). The β-subunits have multiple roles, from modulating channel gating and regulating channel expression, to interacting with the cytoskeleton and the extracellular matrix, as cell adhesion molecules (Brackenbury and Isom, 2008). It is now known that there are nine pore forming α-subunits of sodium channels, Nav1.1-Nav1.9, encoded by genes SCN1A-SCN11A (Catterall et al., 2005), which associate with one or more non-pore-forming β- subunits encoded by SCN1B-SCN4B (Brackenbury and Isom, 2011). In addition to being expressed in cells capable of generating action potentials, sodium channels have also been identified in cells that have not traditionally been considered to be electrically excitable (“non-excitable cells”), leading to speculation as to their functional role (Pappalardo et al., 2016). Sodium channels contribute to multiple, varied cellular functions in these cells including phagocytosis (Carrithers et al., 2007), migration (Kis-Toth et al., 2011), and proliferation (Wu et al., 2006). Voltage-gated sodium channels have been documented in immune cells such as macrophages (Schmidtmayer et al., 1994; Carrithers et al., 2007, 2009, 2011; Black et al., 2013).

Patch-clamp recordings have since confirmed the expression of functional sodium channels in microglia (Korotzer and Cotman, 1992; Nicholson and Randall, 2009; Persson et al., 2014). A number of voltage-gated ion channels have been identified in microglia, in particularly, voltage-gated Na^+^ channels isoforms (VGSC): Nav1.1, Nav1.5, and Nav1.6 (Craner et al., 2005; Black and Waxman, 2012).

*In vitro*, microglia derived from mixed glial cultures from neonatal rats, exhibit immunolabeling for Nav1.1, Nav1.5, and Nav1.6, which is most prominent, while Nav1.2, Nav1.3, Nav1.7, Nav1.8, and Nav1.9 are not detectable above background levels (Black et al., 2009). Whole-cell voltage clamp experiments on cultured rat microglia revealed that, depolarization-induced sodium currents were elicited and then completely blocked by 0.3 μM TTX, consistent with the presence of functional TTX-S sodium channels (Persson et al., 2014). Similarly, microglia within normal CNS tissues exhibit low levels of Nav1.6 immunolabeling *in situ* (Black and Waxman, 2012).

There is a handful of electrophysiological studies of cultures of human microglia derived from native tissue which reports the presence of Na^+^ currents (Nörenberg et al., 1994b; Nicholson and Randall, 2009), however, these are not observed in every laboratory (McLarnon et al., 1997). Na^+^ currents have also been reported in rat microglia (Korotzer and Cotman, 1992). A study in mice provides evidence that Nav1.6, plays a central role in the infiltration and phagocytosis of microglia in experimental autoimmune encephalomyelitis. Furthermore, the same channel is reported to be up-regulated in macrophages and microglia in the lesions of multiple sclerosis patients (Craner et al., 2005). To date there is no direct evidence for the involvement of microglial VGSC in AD. This same group, however, also report the presence of Nav1.1 and Nav1.5 in cultured rat microglia and demonstrate their function in many key microglial processes (Black et al., 2009). Although Aβ is a known activator for microglia, treatment of the human microglial cell line with Aβ (12 h, 10 μM) there was no significant change in Na^+^ current or Nav1.5 expression (Nicholson and Randall, 2009). Although there is clear involvement for VGSC in microglial function its role in AD remain less well defined. This could be due to a number of different contributing factors such as species variation, individual laboratory protocols, as well as non-standardized preparation of exogenous Aβ and Aβ species selection.

## Transient receptor potential channels

Transient receptor potential (TRP) channels are non-selective, non-voltage gated cation channels, ubiquitously expressed in mammalian cells. The TRP gene was initially discovered in Drosophilla where mutant gene expressing animals showed impaired vision due to dysregulated Ca^2+^ influx into photoreceptor cells. TRP channels play important physiological role in cells by their regulation of temperature, chemoception, mechanoception, and nocioception. There are 30 known members of the mammalian superfamily, which can be divided up into six subfamilies, based on amino acid sequence homology. These are: TRPA (Ankyrin); TRPC (Canonical); TRPM (Melastatin); TRPML (Mucolipin); TRPP (Polycystin); and TRPV (Vanilloid). TRP channels are tetramers made of monomeric subunits that include a six trans-membrane (TM) domain with a pore-forming loop between TM 5 and 6. In addition, their C- and N-termini are intracellular. Functionally, they act by changing cytoplasmic free Ca^2+^ concentrations via Ca^2+^ permeable pore or by modulating ionic movement via changes to the membrane potential. Microglia are evidenced to express some TRP subfamily members, including those of the TRPC, TRPM, and TRPV families.

### TRPA

The smallest of the TRP subfamilies. Its only mammalian member is TRPA1, a mechano- and chemo-sensor. Its name is derived from the 14 N-terminal ankyrin repeats. To date there is no evidence that it is present in microglia, although it's silencing in dorsal root ganglion results in reduced microglia activation following hyperalgesia (Meotti et al., 2017). Similarly, there is no evidence of the presence of TRPML nor TRPP channels being expressed in nor influencing function of microglia.

### TRPC

The TRPC subfamily consists of seven homologs (C1-7), with TRPC2 being exclusively expressed in mouse. TRPC members share a structural motif in the COOH-terminal tail, TRP box, located close to the intracellular border of TM6. In addition, they contain three or four N-terminal ankyrin repeats.

TRPC channels are activated via the stimulation of GPCRs and receptor tyrosine kinases, leading to phospholipase C, inositol 1,4,5-triphosphate, and diacylglycerol production. This stimulation results in a biphasic Ca^2+^ release with a first phase ER release, followed by sustained Ca^2+^ influx across the membrane. TRPC channels are known mostly as store operated Ca^2+^ entry (SOCE) mediators.

In microglia, all seven members have shown RNA expression in *in vitro* cell line models, although only C1 and C3 have been reported *in vivo*. TRPC1 is a non-selective Na^+^/Ca^2+^ permeable channel with known function in cell survival and proliferation. Their expression is commonly on organelle membranes such as ER and intracellular vesicles. TRPC1 negatively regulates the ORAI1 Ca^2+^ channel resulting in suppression of NKkB, JNK and ERK1/2 signaling from microglia (Sun et al., 2014).

TRPC3 is widely expressed in the CNS where it has modulation via the growth factor BDNF to induce axonal guidance, neuronal survival, and postsynaptic glutamate transmission. In microglia, pre-treatment with BDNF inhibits NO and TNF-α upregulation, via sustained Ca^2+^ influx through upregulated TRPC3 channels at the plasma membrane. Effects were reversed using the siRNA against TRPC3 (Mizoguchi et al., 2014).

### TRPM

The TRPM subfamily has eight mammalian members. Unlike TRPA/C there are no N-terminus anykin repeats, instead having functional protein domains, in addition to the TRP box, in the C-terminus. TRPM's are non-selective cation channels with a verity of cellular functions including temperature sensing, osmolarity, redox, Mg^2+^ homeostasis, proliferation, and cell death. These channels can be subdivided further into four groups: M1/3; M4/5; M6/7; M2/8. M1, 2, 4, and 7 have all been reported as present in microglia.

TRPM1 was the first to be cloned, in 1998 (Harteneck, 2005), however its function and activation remains unknown. TRPM1 has a high capacitance for splice variance, similarly so with TRPM3-with whom M1 shares strong sequence homology. In murine models of AD (5XFAD/MHCII+) high levels of Aβ plaque burden correlated to an increase in TRPM1 gene expression compared to age matched control animals (Yin et al., 2017).

M2 contains an adapted adenosine 5′-diphosphoribose ribose (ADPR)-recognizing Nudix box domain at its c-terminus. It is a redox modulator, activated by reactive oxygen species, ADP ribose, NAD^+^ and Ca^2+^. M2 will mediate the release of lysosomal Zn^2+^ stores in response to reactive oxygen species, leading to increased cytosolic Zn^2+^ levels, leading to regulation of cell motility and actin remodeling. Additionally, Ca^2+^ influx via TRPM2 leads to increased intracellular insulin release in pancreatic β-cells (Uchida et al., 2011). A number of studies, both *in vitro* and *in vivo* confirm TRPM2 expression and activity in microglia.

In a TRPM2 KO mouse model, microglia show an abolishment of Ca^2+^ influx after LPS or IFNγ stimulation. Activation by these stimuli results in Pyk2-mediated activation of p38 MAPK and JNK signaling as well as an increase in nitric oxide production (Miyake et al., 2014). Similarly studies of MCAO-induced hypoxia in TRPM2 KO mice saw reduced MG activation, reduced cytokine expression and increased brain volume after damage (Huang et al., 2017). Lastly, TRPM2 channels are functionally expressed in the murine microglia cell line BV2. Here these channels have been shown to be involved in LPC-induced p38 MAPK phosphorylation. LPC-induced intracellular Ca^2+^ increase and inward currents dependent on TRPM2 channels (Jeong et al., 2017).

TRPM4 are non-selective cation channels with a greater affinity for Na^2+^ over Ca^2+^. TRPM4 are activated by increased intracellular Ca^2+^ due to changes in cell membrane potential, ATP, PKC-dependent phosphorylation and calmodulin (CaM) binding to the channels C-terminal CaM domain (Nilius et al., 2005). Functional channels were detected in the mouse primary microglia, both quiescent and active. Here they are thought to mediate membrane depolarisation, in correlation to Ca^2+^ influx (Beck et al., 2008). Sulfonylurea receptor 1 activates TRPM4 channels in mouse primary microglia. Receptor binding regulates NOS and NO transcription on microglia activation via LPS action at TLR4 (Kurland et al., 2016).

TRPM7, like TRPM6, is a channel-enzyme. It is Mg^2+^, Zn^2+^, and Ca^2+^ permeable with a strong outward rectifying current-voltage relationship. In addition to its ionic pore, it contains a tyrosine kinase domain on its N-terminal. Activity at both pore region and kinase domain are implemented to be involved in the channels activity. For example, in rat brain microglia there is a strong increase of intracellular Mg^2+^ via the channel, however the currents generated were kinase activity-dependant and not due to pore, nor cell, activation (Jiang X. et al., 2003). TRPM7 also plays a role in cell motility. Migration and invasion of M1 (pro-apoptotic) microglia was observed in rat primary and MLS-9 microglia after priming with LPS (Siddiqui et al., 2014). In addition, flow cytometry and Ca^2+^ imaging studies in neonatal mouse microglia saw an increase in intracellular Ca^2+^ with cell activation by Polyl:C. Increased Ca^2+^ led to a correlated increase in TNFα and P38, in a TRPM7-dependent manner.

### TRPV

The final sub-family are the vanilloids, the largest (1-6) and most in depth studies of the TRP channel families. All TRPVs are highly selective to Ca^2+^. The most well-known is TRPV1 for its actions as a thermosensor (temperatures >43C). V1 mediates heat response and inflammation in addition to nociceptive responses to capsaicin, the main “heat” compound of chili peppers. In addition, application of compounds with a pH < 5.9 will shift the temperature gated threshold of these channels to 20–23 C. Heat-mediated activation is shared quality with other TRPV members, specifically 2, 3, and 4. However, these channels are insensitive to capsaicin and pH. V5 and V6 are not thermosensors but have enhanced selectivity to Ca^2+^ over other monovalent cations. Lastly, all TRPV channels are functionally regulated by their insertion, or retention to the plasma membrane.

TRPV1 has a high protein expression in microglia, with the majority of these channels showing co-localisation to organelles including the golgi, ER, lysosomes, and mitochondria. Interestingly, at resting state there is very little expression at the plasma membrane (Miyake et al., 2015). In a model of rat spinal cord injury, activation of TRPV1 channels, via I.V injection of capsaicin, gave increased expression of SOD1 and pro-inflammatory cytokines from spinal microglia (Talbot et al., 2012). Similar influence on pro-inflammatory markers were observed in retinal microglia where activation of TRPV1 resulted in increased IL-6 and NFkB expression (Sappington and Calkins, 2008). Expression of TRPV1 protein and function was confirmed in HMO6 human microglial cell line. Application of capsaicin resulted in increased intracellular Ca^2+^, and subsequently cytochrome C and cleaved caspase 3 release (Kim et al., 2006). Together this suggests a strong role of TRPV1 in the pro-inflammatory profile of microglia.

Little is known about the other TRPV channels in microglia, although an RNA-based analysis by Raboune et al. (2014). showed upregulation of TRPV1-4 in BV2 cells following cell activation by N-acyl amide.

### Microglial TRP channels in AD

Aβ accumulation, one of the major hallmarks of AD, commonly results in excitotoxicity and cell death via the disruption of normal Ca^2+^ homeostasis and release of pro-inflammatory factors such as ROS, NO, and cytokine release. The previous section highlights the role of TRP channels in intracellular Ca^2+^ regulation as well as differentially switching the phenotype of microglia between M1 (pro-apoptotic) and M2 (pro-survival). Despite this there is little research into glial TRP channel activity in AD, with most of the focus being on neuronal responses.

Aβ treatment of BV-2 cells gave an upregulation of protein and mRNA for TRPC6 that is dependent on NFkB activity. When these cells had TRPC6 knocked down via siRNA, the condition media was neuroprotective to cultured hippocampal cells compared to sham BV2 cells. Neuronal influence of TRPC6 activates via an upregulation of COX2 downstream (Liu et al., 2017). By using familial AD mouse models- APP23 and 5XFAD, plaque associated microglia from these animals were homogenized and run through flow cytometry to observe upregulated genes. From these TRPM1 was pulled out, however its role in AD remains unclear (Yin et al., 2017). BV2 cells treated with either fibrillary or soluble Aβ saw high levels of ROS which was attenuated with simultaneous application of TRPV1 via I-RTX (Schilling and Eder, 2011).

## Calcium channels

Plasma membrane calcium channels are subdivided into three main groups according to their manner of activation; the voltage-gated calcium channels (VGCCs), the store-operating calcium channels (SOCs) and the receptor-operated calcium channels (ROCs). VGCCs specifically, play a vital role in maintaining calcium homeostasis, with important roles in cellular processes such as neurotransmission, control of gene expression, hormone secretion and cell apoptosis (Ertel et al., 2000; Valerie et al., 2013). Therefore, developing therapeutics that target these channels may be of benefit in treating various diseases of the CNS, such as AD. Structurally VGCCs consists of the α1 pore-forming subunit consisting of four transmembrane domains, the cytoplasmic β subunit, the peripheral α2δ and occasionally the γ accessory subunit (Ertel et al., 2000). VGCCs are divided into subfamilies according to their pore-forming subunit; the high voltage-activated channels known as Cav1 (Cav1.1-1.4) and Cav2 (Cav2.1-2.3), and the low voltage-activated Cav3 channels (Cav3.1-3.3) (Ertel et al., 2000).

### Voltage-gated calcium channels

To date, evidence suggesting the existence of microglial VGCCs and their involvement in AD is limited. Although numerous studies, mainly via electrophysiology and Fura-2 calcium imaging, have proven that various agents such as Aβ, ATP, and K^+^, cause an increase in intracellular Ca^2+^, the mechanism by which this phenomenon occurs is still under debate (Korotzer et al., 1995; McLarnon et al., 1999; Valerie et al., 2013). Thus, there is no clear indication of the existence of microglial VGCCs or whether the increase in intracellular Ca^2+^ is due to other factors such as ion exchange transporters or opening of intracellular stores (Korotzer et al., 1995; McLarnon et al., 1999; Valerie et al., 2013).

The majority of human studies, have investigated the presence and functionality of VGCCs in human glioblastoma cell lines, consisting of a mixed culture of glial cells, including astrocytes and microglia. Therefore, a major limitation of human *in vitro* studies, is that identifying VGCCs in glioblastoma cells does not necessarily indicate the presence of these channels in microglia. For instance, Valerie et al. (2013), demonstrated that pharmacological inhibition via the calcium channel blocker (CCB) mibefradil, or siRNA-induced downregulation of the Cav3 channel (T-type current) in human glioblastoma cell lines, led to cell apoptosis. Additionally, Nicoletti et al. (2017) demonstrated that Cav2.1 and Cav2.2 are involved in glial proliferation, through using of pharmacological tools (Nicoletti et al., 2017). Furthermore, via the use of an Iba-1 antibody, a marker of inflammation, and immunohistochemistry, in an *in vivo* rodent glioblastoma model (GL261 glioma cells), it was revealed that the degree of Iba-1 positive microglia had increased following N-type inhibition. This highlights a role of microglial VGCCs not only in cell proliferation and microglial survival, but also in inducing their pro-inflammatory action (Nicoletti et al., 2017). Evidence from human glial cells, demonstrates that VGCCs are expressed in human microglia, and that microglia VGCCs may also have a role in neurotoxicity (Hashioka et al., 2012). Prior to 48-h treatment with LPS and IFN-γ to induce inflammation, primary human microglial cells were treated with the L-type blocker nimodipine, significantly reducing neuronal toxicity induced by the microglia (Hashioka et al., 2012). In contrast to other studies, Hashioka et al. (2012) provided more conclusive evidence in indicating the presence of microglial VGCCs due to the use of primary human microglia and not a cell line consisting of a mixed glial population. A 1999 study demonstrated a more direct involvement of microglia VGCCs with progression of AD by investigating how Aβ_25−35_ alters Ca^2+^ signaling in human microglia (Silei et al., 1999). Incubation with Aβ caused an increase in microglia proliferation and additionally an increase in intracellular Ca^2+^ levels (Silei et al., 1999). As no significant increase in microglial intracellular Ca^2+^ levels were observed when microglia were incubated in Ca^2+^ -free media, it was suggested that this change was due to VGCC-mediated Ca^2+^ influx (Silei et al., 1999). This was verified via co-incubation of microglia with Aβ and the CCBs verapamil, nifedipine and diltiazem which lead to a half-reduction in intracellular levels (Silei et al., 1999). Moreover, incubation of peptide-treated microglia with nifedipine not only lead to a reduction in intracellular Ca^2+^, but also significantly prevented the increase in microglia proliferation induced by the peptide. Therefore, this study proposes that Aβ has the ability to increase microglia number and also induce their activation and consequently inflammatory action, through a VGCC manner (Silei et al., 1999).

In contrast to human studies, the majority of studies using animal models, have not provided conclusive evidence to indicate the existence and activity of VGCCs in microglia (Toescu et al., 1998; Silei et al., 1999).

A possible explanation for this, could be that microglial VGCC expression and activity is species-dependent. For instance, studies have shown that rodent microglia can express very low levels of VGCC activity which may even remain undetected (Toescu et al., 1998). Toescu et al. (1998), demonstrated that adding ATP to microglia isolated from murine cortex lead to a significant increase in intracellular Ca^2+^ levels. In contrast, KCl induced microglial depolarisation, did not lead to an increase in intracellular Ca^2+^ thus it was proposed that increased Ca^2+^ levels involved VGCC independent pathway (Toescu et al., 1998). Prolonged elevation in intracellular Ca^2+^ levels can activate pathways involved in regulation of gene expression such as the Ca^2+^-calmodulin pathway, and therefore altered Ca^2+^ signaling in microglia may occur as a pathway for microglia activation and may even induce the progression of various pathological conditions such as AD (Toescu et al., 1998).

Although the majority of animal model studies have not definitively proven the existence of the channels in microglia, a few were able to provide some evidence indicating their existence. In a study carried out in 2014 by Saeugusa and Tanabe, where rodent lines were created where expression of Cav2.2 was suppressed, they indicated dynamic modulation of microglia Cav2.2 in regulation of pain related behavior. (Saegusa and Tanabe, 2014). Saeugusa and Tanabe also highlight neuronal and microglial crosstalk, in controlling response to pathology (Saegusa and Tanabe, 2014). A more recent study investigated how microglial activation, verified by immunostaining and morphological changes, alters the activity of the L-type currents in an *in vivo* animal model for neurodegeneration, and in the *in vitro* BV2 cell line (Espinosa-Parrilla et al., 2015). Comparison of microglia before and after LPS and IFNγ stimulation revealed differences as seen via immunostaining and molecular approaches such as western blotting and PCR (Espinosa-Parrilla et al., 2015). Additionally, as depolarisation of LPS/IFNγ treated microglia demonstrated changes in intracellular Ca^2+^ by treatment with either nifedipine or Bay K8644 (agonist), it was suggested that VGCCs, may form part of the mechanism involved in the activation of microglia, inducing their pro-inflammatory action (Espinosa-Parrilla et al., 2015).

To summarize, even though human microglia studies have proposed the existence of functional VGCCs, the majority of the studies were carried out in mixed glial cell lines. Additionally, animal studies have either demonstrated very low expression of VGCCs in microglia or were not able to prove their existence, either at a functional or expression level (protein and mRNA). Thus, due to the limited and contradicting evidence on human and rodent microglial VGCC existence, the use of human induced pluripotent stem cells (iPSCs) may allow a more effective study of microglial ion channel role in neuro-inflammation observed in neurodegenerative diseases such as AD.

## Chloride channels

Chloride channels are a diverse superfamily of channels proteins, incorporating the volume regulated chloride channels, the ClC proteins, Ca^2+^ activated chloride channels, CFTR and maxi chloride channels (Alexander et al., 2017). Studies have identified Cl^−^ channels in rat (Visentin et al., 1995; Schlichter et al., 1996), bovine (McLarnon et al., 1995) and human microglia (McLarnon et al., 1997). These have mainly been based on pharmacological studies using a range of Cl^−^ channel blockers (e.g. nifluemic acid). Pharmacological modulation of Cl- channels indicates a role for in the regulation of microglia process outgrowth (Hines et al., 2009). However, the lack of specific pharmacological tools has hindered our progress in identifying specific channel entities, and indeed their contribution to microglia physiology. This is backed up with a lack of experimental evidence as to the molecular identity of the channels that have been suggested to be responsible for experimental observations. While the molecular identity remains to be resolved, evidence indicates that is a similar fashion to Cl^−^ currents within lymphocytes, microglia Cl^−^ conductance are responsive to stretch (Lewis et al., 1993; Steinert and Grissmer, 1997). Interestingly CLIC1 an intracellular chloride channel has received some attention with relation to amyloid pathology (Novarino et al., 2004; Milton et al., 2008; Paradisi et al., 2008). This suggests a role for modulation of chloride conductances in microglial generation of reactive oxygen species, but robust evidence for this is lacking in relevant *in situ* models of microglia.

## Voltage gated proton channels (HV1)

Voltage-gated proton channels (H_v_1; Alexander et al., 2017) reportedly consist of 4 proton sensitive transmembrane domains which are sensitive to both membrane depolarisation and transmembrane pH gradient (DeCoursey, 2008; Capasso et al., 2011). There is widespread expression of these channels within the central nervous system, highlighting both regional and cellular variation (Eder et al., 1995; McLarnon et al., 1997). Functional evidence comes from both studies carried out on murine microglia (Eder et al., 1995; Klee et al., 1998, 1999), rat (Visentin et al., 1995), and human microglia (McLarnon et al., 1997). There is also evidence to link Hv1 to both microglia polarity and brain responses to stroke (Wu et al., 2012; Tian et al., 2016). This could be pertinent given the link between hypoxia and Alzheimer's disease (Peers et al., 2007). However, one drawback from these studies is the use of culture preparations. This is pertinent given that work on brain slices was unable to detect any H^+^ conductance *in situ* (De Simoni et al., 2008). This again raises the question about membrane properties in cultured preparations in contrast to *in situ* set ups. In addition there are questions around the physiological role of these channels when present in microglia (Eder and Decoursey, 2001) It is well established that microglial reactive oxygen species contribute to neuronal cell death in AD (see review Block et al., 2007). This process likely involved the build-up of protons within microglia, which will impact on the flux through Hv1 channels. However, a direct demonstration of the involvement of H_V_1 in this process is lacking. There is greater evidence to indicate the involvement of other channels (e.g., Kv1.3) which are discussed elsewhere in this article.

## Modeling microglial involvement

To fully understand a disease and its etiology it is necessary that extensive modeling takes place. By tradition this has been through the use of a number of different model systems including both animal (murine) models and primary patient cell lines. Currently in AD research there is a large focus on the use of animal models, particularly transgenic mice (McGowan et al., 2006), as a lot is understood about their genetics and the availability of well-characterized genetic manipulation techniques in this organism. Not only this, mice are more closely phylo-genetically related to humans than other model systems such as *Caenorhabiditis elegans* or *Drosophila melanogaster*. The genetic similarities between humans and mice means that they have utility in studying the familial aspect of AD by using transgenic mice that contain mutations in the APP and PSEN genes. There are over 100 different transgenic mouse models available to study the familial aspect of AD, with some models containing five different mutations in the APP and PSEN genes (Oakley et al., 2006).

As it is widely accepted that Aβ plaques and neurofibrillary tangles cause neuro-inflammation, models which overexpress mutant human versions of APP have been shown to present microglial activation (Bornemann et al., 2001; Wright et al., 2013). In addition to this it was shown that there were significant increases in CD38-positive microglia before Aβ deposition which also correlated with neuronal cell death in the CA1 region of the hippocampus (Wright et al., 2013). The inflammatory processes of in another APP mouse model, Tg2576, were investigated by looking at individual microglial cells using *in vivo* multiphoton imaging. Meyer-Luehmann and his colleagues showed that Aβ plaques can form within days and once formed it only takes 1–2 days before microglial cells begin to aggregate around the depositions. Alongside this accumulation of microglia is accompanied by changes to neurite morphology (Meyer-Luehmann et al., 2008). Whilst these have their advantages, they also have a number of limitations. These types of models do not accurately recapitulate human pathology as they do not develop the robust tauopathy or neuronal cell death that is seen in human disease without the addition of extra transgenes such as tau (Ribé et al., 2005).

The triple-transgenic model of AD, which contains the APP_SWE_, Presenilin-1 (PSEN_M146V_) and tau mutations (tau_P301L_) offers the advantage that they develop Aβ plaques, tau tangles, synaptic dysfunction and LTP deficits which all manifest in an age-related manner (Oddo et al., 2003). Janelsins et al. demonstrated that this model shows a 14.8 fold increase of TNF-α and 10.8 fold increase in MCP-1 mRNA in 6 month old triple transgenic mice when compared to 2 month old mice. However these increases were only seen in the entorhinal cortex and could not be replicated in the hippocampus, suggesting that different cell types or environments may be responsible for the differential transcript levels and inflammatory responses in these disease relevant brain regions (Janelsins et al., 2005).

Mouse models containing just tau mutations have also been investigated in terms of neuro-inflammatory response and they too also display microglial changes (Wes et al., 2014; Cook et al., 2015). For example, the P301S tau model whose neurons develop bundles of hyperphosphorylated tau also have significant increases in inflammatory molecules such as IL-1β and COX-2 within the tau-positive neurons. Alongside this they also demonstrated that there were activated microglia throughout the brain and spinal cord, but that these microglia could be predominantly found surrounding the tau-positive neurons (Bellucci et al., 2004). Interestingly, this microglial activation was shown to begin before neurofibrillary tangle formation, but could be ameliorated using an immunosuppressive drug, FK506, early in life increasing life span and attenuating tau pathology (Yoshiyama et al., 2007). One important thing to bear in mind that mutations in tau do not cause AD but instead cause frontotemporal dementia. So whilst these models can provide useful information about how mutations in tau can cause cellular dysfunction and neurodegeneration they do not completely replicate AD in terms of other pathological markers (Wolfe, 2012).

Whilst proven useful for modeling autosomal disease, such as the familial form of AD, as previously mentioned, these murine models do not accurately recapitulate AD. A more promising avenue for modeling complex diseases, such as sporadic AD, is through the use of stem cell technology. Embryonic stem cells (ESCs) are derived from the inner cell mass, or blastocyst, of an embryo and can differentiate into any cell in the body (Evans and Kaufman, 1981). Despite their many potential uses, the ethical issues surrounding the use of embryo-derived cells are numerous. However, recent advances in stem cell technology have meant that it is now possible to derive stem cells from differentiated adult cells/tissue. Takahashi et al. showed it was possible to use ectopic transcription factors to induce pluripotency and ESC properties (Takahashi and Yamanaka, 2006). These transcription factors were known for being important in the long term maintenance of ES cell phenotype (Oct3/4 and Sox2) and pluripotency (c-myc and Klf4) (Takahashi and Yamanaka, 2006). These iPSCs are almost identical to ESCs in terms of their characteristics. They are able to differentiate into any cell type in the body, have infinite potential to grow, share the same morphology and have the same expression pattern of genes (Yamanaka, 2009); making them a potentially very powerful tool for complex disease research.

Primary microglia cultures are often used to study neuro-inflammation, they can be derived from rat or mouse brain before birth or early on in development. In addition, human microglia cultures have also been established from fetal brain (McLarnon et al., 1997). One method of generating these cells was developed by Giulian and Baker (1986) and involving a specific process of adhesion and agitation. These cells are often used as they show similarities to microglial cells *in vitro*, however, the process of extraction and culture itself alters microglial phenotype (Caldeira et al., 2014). Given the degree of variability in ion channel distribution during development and aging (Harry, 2013), using this type of model for investigating neurodegeneration is less than ideal. Another method to study microglia is through the use of retroviral-immortalized cell lines, such as the mouse and rat microglial cell lines N9 and BV-2 respectively (Righi et al., 1989; Blasi et al., 1990). These cell lines offer an advantage in the fact that they are fast to grow, and large numbers of cells can be generated quickly. However, as they have been immortalized using oncogenes which means they differ from primary microglia as they have increased adhesion and proliferation and can vary in terms of their morphology (Horvath et al., 2008).

Until recently, being able to generate iPSC-derived microglia has been elusive, with previous attempts being met with skepticism as the microglia were made from induced hematopoietic stem cells (HSCs). HSCs have the potential to give rise to other cell types such as blood derived macrophages and as already stated microglia arise from EMPs. In order to generate EMPs from the iPSCs, Muffat and his colleagues developed a serum free media that contains high levels of IL-34 and colony stimulating factor 1 (CSF1) (Muffat and Li, 2016). These conditions were chosen as the media mimics the brain cerebrospinal fluid and the factors have been shown to be necessary for microglia differentiation and maintenance. Under these conditions they found that the cells soon formed rope-like structures that when plated onto low adherence plates gave rise to highly adherent pluripotent stem cell–derived microglia-like cells (pMGLs). These cells express many of the markers that would be expected from microglia, such as TMEM119, P2RY12/13, HEXB and GPR34. Alongside this they are also highly phagocytic and gene transcriptomic analysis demonstrated they resemble human primary fetal and adult microglia (Muffat and Li, 2016). This is not the only protocol that has been published which describes the derivation of microglia from iPSCs, subsequently there have been four more protocols released. In fact the next two papers described the generation of iPSC-derived microglia going through a hematopoietic progenitor cell (HPC) stage. Both methodologies use defined media systems that contain a number of growth factors including IL-3, BMP4 and L-ascorbic acid to generate HPCs (Abud et al., 2017; Pandya et al., 2017). Both protocols take about 10 days to generate HPCs at which point they were checked for markers of the hematopoietic lineage such as CD43 before differentiating for a further 2 weeks using another media to form induced microglia like cells (iMGLs). One way in which these protocols differ is that Pandya et al. (2017) co-culture the HPCs with astrocytes to enhance microglial differentiation. This is not the only protocol that uses co-culture to generate iPSC-derived microglia, a paper released by Walter Haenseler also uses co-culture but neuronal microglial co-culture instead (Haenseler et al., 2017).

Whilst iPSC-based models offer a number of advantages to modeling complex diseases there are a number of limitations that should also be considered. Firstly, the cells which are derived from iPSCs have been found to display the functional and epigenetic signatures of fetal neurons and do not maintain the features, such as telomere length and mitochondrial metabolism, of the cells from which they were originally derived (Lapasset et al., 2011). One of the current major stumbling blocks for iPSC research (control and those derived from patients with specific neurodegenerative disorders), is the lack of standard culturing or differentiation methods (Wen et al., 2016). Resulting in the unavailability of established protocols to generate entirely pure populations of a specific cell type, therefore making cross lab comparisons particularly difficult. However, more recently the availability of human tissue as well as iPSCs have provided new opportunities for academic and industry-based researchers to identify optimal cell types and culture conditions to efficiently generate stable, defined and reproducible cell types for their specific research–with limited variability. Whilst this may not be an issue for some studies, when trying to investigate diseases of aging such as AD it could pose more problems as cells may not show age related phenotypes or degeneration. One way in which it may be possible to overcome this is through maintain and aging the cells in culture for as long as possible.

One of the challenges to date has been modeling sporadic AD in both rodent and human models of disease, with familial AD mutations accounting for only 5–10% of all AD cases (Kim et al., 2017). Excitingly, Lin et al. (2018) describes the first experiments in which CRISPR/Cas9 technology has been used to generate isogenic APOE4 iPSC-derived microglia. In this study the APOE4-like microglia exhibited altered morphology correlating to the reduced Aβ phagocytosis seen in rodent models. They found that consistently converting APOE4 to APOE3 in brain cell types from sporadic AD iPSCs was sufficient to diminish multiple AD-related pathologies (Lin et al., 2018). They also showed that in their iPSC-derived microglia, TREM2 was positively correlated to the APOE4 genotype. This data is consistent with reports showing increased levels of soluble TREM2 in cerebrospinal fluid of AD patients (Heslegrave et al., 2016). Similarly, protocols for microglia differentiated from patients carrying missense mutations in TREM2 (that are causal for frontotemporal dementia-like syndrome and Nasu-Hakola disease). These studies found subtle effects on microglia biology, consistent with the adult onset of disease in individuals with these mutations (Brownjohn et al., 2018). These particular studies establish a reference for human cell-type-specific changes associated with the risk of developing AD, providing critical insight into potential treatments for sporadic AD.

As more is understood about the developmental origin and unique identity of microglia, recent studies have attempted to circumvent this issue by deriving microglia from iPSCs in order to study human and cell-type-specific biology and disease (Muffat et al., 2016; Abud et al., 2017; Douvaras et al., 2017; Haenseler et al., 2017; Pandya et al., 2017; Takata et al., 2017; Brownjohn et al., 2018; Lin et al., 2018). At the whole-transcriptome level, microglia generated by the methods reported here most closely resemble cultured primary microglia (Brownjohn et al., 2018). Due to a lack of unique surface markers, it has historically been difficult to distinguish microglia from other macrophages and cells of myeloid lineage. It is only recently that a distinct transcriptomic profile of microglia has emerged (Hickman et al., 2013; Butovsky et al., 2014; Holtman et al., 2015; Bennett et al., 2016; Gosselin et al., 2017; Keren-Shaul et al., 2017; Krasemann et al., 2017). In this review we have highlighted the similarities between rodent and human microglia transcriptomics and have identified key ion channels prominent in human iPSC-derived microglia, some of which we have already been highlighted earlier in this review as prominent targets associated with AD (Table 2) including KCNK13, KCNN4, TRPV2, HVCN1, and CLIC1. Indeed, the ion channels found from iPSC-derived microglia to date mirror those found in aged-human tissue (Olah et al., 2018).

**Table 2:** Comparison of multiple transcriptome studies of regulated microglial genes, relating to ion channels, in models of aging or Alzheimer's disease.

**Species**	**Sample type**	**Potassium channels**	**Sodium channels**	**TRP channels**	**Calcium channels**	**Others**	**Reference**
Human	iPSC	KCNA5, KCNK13, KCNN4	SCN5A	TRPM2, TRPM4, TRPM8, TRPV1, TRPV2	CACNA1S	HVCN1, CLIC1	Haenseler et al., 2017
Human	Biopsy primary microglia culture	KCNK13, KCNN1, KCNN4	Not determined	TRPC1, TRPC2, TRPM2, TRPM3, TRPM4, TRPM7, TRPMV1, TRPV2, TRPV4	Not determined	HVCN1, CLIC1	Gosselin et al., 2017
Human	Purified from post-mortem dorsal lateral pre-frontal cortex	KCNN4	Not determined	TRPM2, TRPV2	Not determined	CLIC1	Olah et al., 2018
Human	Purified from post-mortem dorsal lateral pre-frontal cortex	KCNJ2, KCNK13, KCNN4	Not determined	TRPM2, TRPM7, TRPV1, TRPV2	CACNA1A, CACNA1D	HVCN1, CLIC1	Olah et al., 2018
Human	Purified from post-mortem right parietal cortex	KCNK13, KCNN4	Not determined	TRPC2, TRPV2, TRPV4	Not determined	CLIC1	Galatro et al., 2017
Human	Purified from post-mortem right parietal cortex	KCNN4	Not determined	Not determined	CACNA1F	Not determined	Galatro et al., 2017
Mouse	Primary microglia culture	KCNA3, KCNK13, KCNN4	Not determined	TRPM4	CACNA1A, CACNA1D	Not determined	Gosselin et al., 2017
Mouse	Collated Meta-Analysis	KCNA1, KCNA2, KCNN1, KCNN3	Not determined	TRPA1, TRPC1, TRPC3, TRPC4, TRPC6, TRPC7, TRPM3, TRPM8, TRPV1, TRPV6	Not determined	HVCN1	Olah et al., 2018
Rat	Primary microglia culture	KCNK13, KCNN4	Not determined	TRPC4, TRPC6, TRPM2, TRPM4, TRPV1	Not determined	CLIC1	Bohlen et al., 2017

*Regulation threshold was set at a 3-fold change over all studies. Anything below this is referred to as not determined*.

Finally, the characterization of the electrophysiological properties of neurons derived from iPSCs are extremely limited and even fewer reports on the functional properties of iPSC-derived glia (microglia and astrocytes). However, with the development of standardized methods and differentiation protocols and, importantly, broader *functional* characterization of the complex collection of ion channels and receptors expressed in defined glial and neuronal subtypes from iPSCs, their significance in drug discovery and neuroscience will become increasingly valuable.

## Concluding remarks

Microglial research has expanded dramatically in the last 5 years, this combined with the lack of new therapeutic options for treating complex neurological conditions highlights the potential of these cells to provide a viable alternative. For this to be realized a clearer picture of human microglial physiology needs to be established. The development of iPSC technology has been a great advance in these efforts, but robust protocols are still in their infancy (Douvaras et al., 2017; Haenseler et al., 2017; Brownjohn et al., 2018). With microglia being dependent *in situ* environments the need to generate more complex 3D models is even greater. While the development of 3D scaffolds continues at pace (Saliba et al., 2018), some initial research indicates the possibility of 3D microglia cultures (Cho et al., 2018). The challenge will now be to incorporate the diverse range of cells into these cultures with the ability to provided measurable outcomes (e.g. electrophysiology). Establishing robust and reproducible protocols will also allow us to progress into addressing the role of microglia in pathological states. This is vital if we are to achieve a therapeutic purpose for targeting microglia ion channels.

The role of ion channels is extensive within the central nervous system, however as non-excitable cells microglia channels often get overlooked. Here we have examined the microglia ion channel landscape and the evidence that supports the involvement in Alzheimer's disease pathogenesis. While there is still work to be done as highlighted above, this review indicates that microglia ion channels play a pivotal role in their physiology and can contribute to the fight against dementia.

## Author contributions

TK and MD contributed to the initial design and conception of the review. LT, JI, EK, MD, and TK wrote individual sections of the review. TK wrote the first draft of the manuscript. LT and JI prepared figures. TK and LT outlined the tables and compiled final version of manuscript. All authors approved, read and revised final version before submission.

### Conflict of interest statement

The authors declare that the research was conducted in the absence of any commercial or financial relationships that could be construed as a potential conflict of interest.
